# Supplementary figures and images for: Analysis of residual disease in periocular basal cell carcinoma following hedgehog pathway inhibition: Follow up to the VISORB trial
Source: PLoS One. 2022 Dec 1;17(12):e0265212. doi: 10.1371/journal.pone.0265212 (PMC9714843; doi:10.1371/journal.pone.0265212)

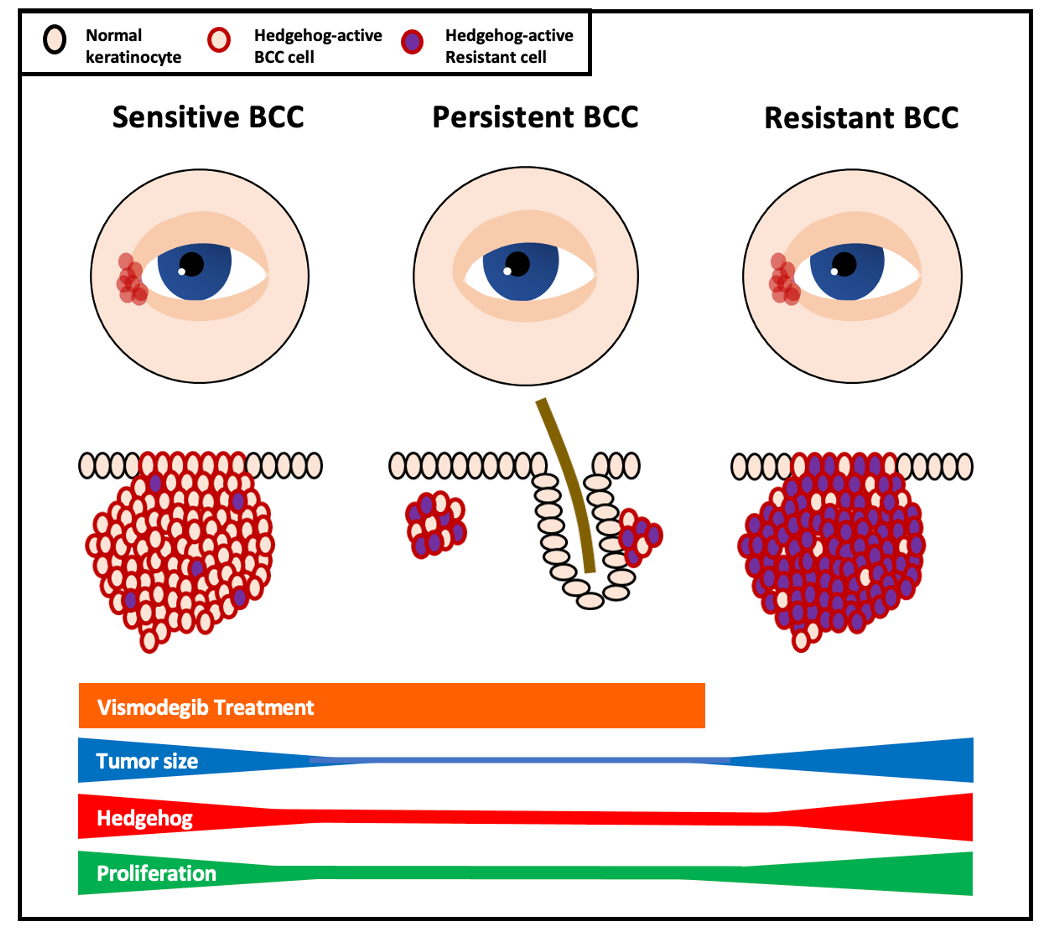

Supplement: S1 Graphical abstract — (TIF) [file pone.0265212.s001.tif]
