# Supplementary material for: Analysis of residual disease in periocular basal cell carcinoma following hedgehog pathway inhibition: Follow up to the VISORB trial
Source: PLoS One. 2022 Dec 1;17(12):e0265212. doi: 10.1371/journal.pone.0265212 (PMC9714843; doi:10.1371/journal.pone.0265212)
Supplement: Attachment — Submitted filename: PLos one review .docx [file pone.0265212.s003.docx]

The result show one of the first studies that describe pathogenic variants of PTCH1, and SMO . The authors further haracterized each variant using the VarSome.com database . These kind of multi center studies offers to the literature and most importantly to clinicians a better knowledge and an inspiration for further investigations,
